# Dietary encapsulated probiotic effect on broiler serum biochemical parameters

**DOI:** 10.14202/vetworld.2018.1344-1348

**Published:** 2018-09-29

**Authors:** P. Yazhini, P. Visha, P. Selvaraj, P. Vasanthakumar, V. Chandran

**Affiliations:** 1 Suguna Institute of Poultry Management, Udumalpet, Tirupur, Tamil Nadu, India; 2 Department of Veterinary Physiology, Veterinary College and Research Institute, Namakkal, Tamil Nadu, India; 3 Faculty Veterinary University Training and Research Centre, Karur, Tamil Nadu, India

**Keywords:** biochemical, broiler, encapsulated, probiotic, serum

## Abstract

**Aim:**

The study aimed to evaluate the effect of encapsulated probiotic bacteria (*Lactobacillus lactis* and *Bifidobacterium bifidum*) on broiler serum biochemical parameters.

**Materials and Methods:**

Encapsulation protects the probiotics and increases their livability on exposure to unfavorable processing and storage temperatures and gastrointestinal pH. Hence, an *in vitro* study was undertaken to encapsulate the probiotic bacteria *L. lactis* and *B. bifidum* with sodium alginate and chitosan and evaluate the encapsulation efficiency. This experiment was conducted with 288-day-old broiler chicken; they were distributed randomly into eight treatments and six replicates in each treatment (six birds in each replicate) and given with standard feed.

**Results:**

Supplementation of the encapsulated bacteria either alone or in combination (T_4_, T_6_, and T_8_) significantly (p<0.05) increased mean total serum protein, albumin, and globulin as compared to the birds that were not supplemented with any probiotic (T_1_ and T_2_) or supplemented with non-encapsulated bacteria (T_3_, T_5_, and T_7_). Supplementation of the encapsulated bacteria either alone or in combination (T_4_, T_6_, and T_8_) significantly (p<0.05) lowered mean total serum cholesterol, serum low-density lipoprotein (LDL) cholesterol, and serum triglycerides, as compared to the birds that were not supplemented with any probiotic (T_1_ and T_2_) or supplemented with non-encapsulated bacteria (T_3_, T_5_, and T_7_).

**Conclusion:**

It may be concluded that supplementation of the encapsulated probiotic bacteria either alone or in combination significantly increased total serum protein, albumin, and globulin and significantly lowered mean total serum cholesterol, serum LDL cholesterol, and serum triglycerides as compared to the birds that were not supplemented with any probiotic or supplemented with non-encapsulated bacteria.

## Introduction

Along with the increase in demand for quality of animal product, concerns about the effects of these products on human health are also increasing. Hence, the focus should be made not only on high productivity but also on their impact on human health and the environment. Even though antibiotics are shown to increase production in broiler industries, focus on antibiotic resistant also increased. To provide good quality broiler meat without compromising, the production level probiotics are shown to be the best way. Probiotics can be defined as live microorganisms which, when administrated in adequate numbers, confer health benefits to the host by improving the microbial balance [1,2]. Many research studies have reported that inclusion of probiotic species such as *Lactobacillus, Streptococcus, Bacillus, Bifidobacterium, Enterococcus, Aspergillus, Candida*, and *Saccharomyces* in broiler nutrition has a beneficial effect on growth performance, intestinal health, immune status, and meat characteristics such as microbial load, keeping quality, and sensory evaluation [3].

While supplementing probiotics to birds and animals, activity and stability of probiotic microorganism are affected by different storage temperature, stability in dried and frozen form, acidic and alkaline pH of gastrointestinal tract, [4]. Many reports have indicated that there is poor survival of bacteria in products containing free probiotic cells during passage through the upper gastrointestinal system [5].

Encapsulation is the process which enhances the survivability and stability of probiotic bacteria. There are various methods employed in encapsulation among them encapsulation of probiotic bacteria with alginate and chitosan provides protection in simulated gastrointestinal condition, and therefore, it is a good way of delivering viable bacteria cells to the intestine [6].

The study aimed to encapsulate the probiotic bacteria (*Lactobacillus lactis* and *Bifidobacter bifidum*) for supplementation through feed and to evaluate the effect of encapsulated probiotic bacteria on broiler serum biochemical index.

## Materials and Methods

### Ethical approval

This study was approved by Tamil Nadu Veterinary and Animal Sciences University Ethical Committee.

### Encapsulation

Encapsulation of *Lactobacillus lactis* and *Bifidobacterium bifidum* was carried out separately following the method of Sharma *et al*. [7] with slight modifications. 100 mg of probiotic culture (*L. lactis*/*B. bifidum*) was inoculated in 5 mL of specific broth DeMan Rogosa and Sharpe (MRS) broth for *L. lactis* and *Bifidobacterium* broth for *B. bifidum*) and incubated anaerobically at 37°C for overnight. After incubation, the culture was centrifuged at 4000 rpm for 30 min, and the supernatant was removed. The bacterial pellet was washed thrice with phosphate-buffered saline (PBS) (pH - 7.0), resuspended in 1 mL PBS, and then mixed with 10 mL of 4% autoclaved sodium alginate solution. This mixture was extruded using a sterile insulin syringe into 100 mL of gently stirred autoclaved 2.5% calcium chloride solution with the help of a magnetic stirrer. The distance between the tip of the syringe and CaCl_2_ solution was 30 cm. The droplets formed gel spheres instantaneously. The beads were left in the hardening solution for 30 min and then transferred into 100 mL of the coating solution (autoclaved 0.4% chitosan solution) and left for 30 min with constant stirring. The dried beads were stored in sterile vials until further use (biological trial).

### Experimental birds and diet

A total of 288 numbers of day-old broiler chicks were wing banded, weighed, and randomly allotted to eight groups with six replicates of six chicks each based on the body weight with all replicates having similar body weight. The dietary supplementation of non-encapsulated and encapsulated bacteria (*L*. *lactis* and *B*. *bifidum)* followed as mentioned below.

The treatment groups consisted of basal diet T_1_ (basal diet with antibiotic), T_2_ (basal diet without antibiotic), T_3_ (basal diet + non-encapsulated *L*. *lactis* 1×10^9^ cfu/kg feed), T_4_ (basal diet + encapsulated *L. lactis* 1×10^9^ cfu/kg feed), T_5_ (basal diet + non-encapsulated *B. bifidum* 1×10^12^ cfu/kg feed), T_6_ (basal diet + encapsulated *B. bifidum* 1×10^12^ cfu/kg feed), T_7_ (basal diet + non-encapsulated *L*. *lactis* 1×10^4^ cfu/kg of feed and *B*. *bifidum* 1×10^6^ cfu/kg feed), and T_8_ (basal diet + encapsulated *L. lactis* 1×10^4^ cfu/kg feed and *B*. *bifidum* 1×10^6^ cfu/kg feed). The probiotic-supplemented groups were fed basal diets without any antibiotic.

### Blood collection

At the end of 4^th^, 5^th^, and 6^th^ week of age, 5 mL blood samples were collected using 23G sterile needles from wing vein of six birds per treatment. Plasma samples for the estimation of biochemical constituents were obtained by collecting blood in sterile tubes with ethylenediaminetetraacetic acid (EDTA) as an anticoagulant. Serum samples for hemagglutination titer assessment were obtained by collecting blood in sterile tubes without EDTA.

### Estimation of blood parameters

The blood biochemicals were analyzed using commercial kits (Span Diagnostics Ltd., India) in UV-visible double beam spectrophotometer (SYSTRONICS, Model 2202, India). Total cholesterol content, high-density lipoprotein (HDL) cholesterol [8], glucose [9], triglycerides [10], total protein [11], albumin, and globulin [9] content were estimated in the plasma.

### Statistical analysis

The data collected on various parameters were grouped and subjected to statistical analysis by one-way ANOVA using SPSS, version 20.0 for Windows (IBM, USA).

## Results

### Effect on total proteins, albumin, and globulin

The effect of supplementation of non-encapsulated and encapsulated probiotic bacteria alone and their combination on the mean total proteins, albumin, and globulin (g/dL) in broiler chicken is presented in Table-1.

**Table-1:** Mean (±S.E.) plasma protein profile (g/dL) of broiler chickens supplemented with non-encapsulated and encapsulated probiotic bacteria.

Treatment groups	Total proteins		Albumin		Globulin	
		
	4^th^ week	6^th^ week	4^th^ week	6^th^ week	4^th^ week	6^th^ week
T_1_ - basal diet+50 mg oxytetracycline/kg	4.40^a^±0.2	4.47^a^±0.1	1.80^a^±0.3	2.01^a^±0.3	2.61^a^±0.3	2.46^a^±0.4
T_2_ - basal diet+(without antibiotic)	4.24^a^±0.4	4.43^a^±0.9	1.69^a^±0.4	2.00^a^±0.4	2.55^a^±0.9	2.23^a^±0.4
T_3_-T_2_+non-encapsulated *Lactobacillus lactis*	5.15^b^±0.4	5.49^b^±0.1	1.87^b^±0.3	2.13^b^±0.4	3.27^b^±0.5	3.35^b^±0.3
T_4_-T_2_+encapsulated *Lactobacillus lactis*	5.81^c^±0.1	6.19^c^±0.2	2.16^c^±0.4	2.25^cd^±0.2	3.72^d^±0.3	3.92^c^±0.2
T_5_-T_2_+non-encapsulated *Bifidobacterium bifidum*	5.08^b^±0.5	5.59^b^±0.8	1.98^b^±0.4	2.12^b^±0.4	3.11^b^±0.2	3.47^b^±0.8
T_6_-T_2_+encapsulated *Bifidobacterium bifidum*	5.94^c^±0.2	6.11^c^±0.1	2.26^c^±0.3	2.26^cd^±0.2	3.79^d^±0.2	3.85^c^±0.2
T_7_-T_2_+non-encapsulated *Lactobacillus lactis+Bifidobacterium bifidum*	5.19^b^±0.4	5.69^b^±0.3	2.00^b^±0.4	2.19^b^±0.4	3.18^b^±0.4	3.52^b^±0.5
T_8_-T_2_+encapsulated *Lactobacillus lactis+Bifidobacterium bifidum*	6.05^d^±0.3	6.34^c^±0.3	2.26^c^±0.3	2.35^d^±0.2	3.81^d^±0.3	3.99^c^±0.5

Means within the same column bearing different superscripts differ significantly (p<0.05)

The mean plasma total protein values of T_1_ and T_2_ were similar and significantly (p<0.05) lower as compared to all other treatment groups. The mean plasma total protein values between T_4_, T_6_, and T_8_ were similar and significantly (p<0.05) higher than all the other treatment groups. There was no significant difference in mean protein values among T_3_, T_5_, and T_7_ groups.

The mean plasma albumin value was significantly (p<0.05) lowest in T_1_ and T_2_ groups as compared to all the other treatment groups. T_8_ group of birds showed significantly (p<0.05) higher mean plasma albumin values. There was no significant (p>0.05) difference among plasma albumin values of birds belonging to T_3_, T_5_, and T_7_ and between T_4_ and T_6_.

The mean plasma globulin value was significantly (p<0.05) lowest in T_1_ and T_2_ groups of broiler chicken in comparison to all the other treatment’s groups. There was no significant (p>0.05) difference in the plasma albumin values of birds belonging to T_3_, T_5_, and T_7_. T_4_, T_6_, and T_8_ groups of birds showed significantly (p>0.05) higher mean plasma globulin values as compared to the other treatment groups.

### Effect on plasma lipid profile

The effect of supplementation of non-encapsulated and encapsulated probiotic bacteria alone and their combination on the mean lipid profile in the broiler chicken is presented in Table-2.

**Table-2:** Mean (± SE) lipid-protein profile (mg/dl) of broiler chicken supplemented with non-encapsulated and encapsulated probiotic bacteria.

Treatment groups	Total cholesterol	HDL cholesterol	LDL cholesterol	Triglycerides
T_1_ - basal diet+50 mg oxytetracycline/kg	198.3^e^±1.1	67.7^a^±0.2	111.2^d^±1.3	103^e^±0.3
T_2_ - basal diet+(without antibiotic)	199.5^e^±2.3	66.6^a^±1.1	112.1^d^±2.1	104^e^±0.1
T_3_-T_2_+non-encapsulated *Lactobacillus lactis*	184^cd^±0.2	70.8^b^±0.3	93.44^c^±4.2	98.8^d^±0.1
T_4_-T_2_+encapsulated *Lactobacillus lactis*	179.3^b^±0.1	77.7^c^±0.2	84.9^b^±0.2	87.6^b^±0.1
T_5_-T_2_+non-encapsulated *Bifidobacterium bifidum*	186.8^d^±0.4	71.2^b^±0.2	95.88^c^±2.7	98.6^d^±0.1
T_6_-T_2_+encapsulated *Bifidobacterium bifidum*	179.6^b^±0.2	77.6^c^±0.3	82.48^b^±0.4	87.6^b^±0.1
T_7_-T_2_+non-encapsulated *Lactobacillus lactis+Bifidobacterium bifidum*	181^bc^±0.1	76.4^c^±1.0	85.58^c^±4.8	95.1^c^±0.3
T_8_-T_2_+encapsulated *Lactobacillus lactis+Bifidobacterium bifidum*	171.4^a^±2.5	80.6^d^±0.1	73.7^a^±2.4	85.5^a^±0.1

Means within the same column bearing different superscripts differ significantly (p<0.05), HDL=High-density lipoprotein, LDL=Low-density lipoprotein

### Plasma total cholesterol, low-density lipoprotein (LDL) cholesterol, and HDL cholesterol

The mean plasma total cholesterol levels were significantly (p<0.05) highest in T_1_ and T_2_ groups of broilers when compared with all other treatment groups. Among probiotic-supplemented groups, the mean plasma cholesterol level was significantly (p<0.05) lower in the T8 group. In general, encapsulated probiotic-supplemented group had significantly (p<0.05) lower mean plasma total cholesterol as compared to the birds supplemented with non-encapsulated probiotic-supplemented groups.

The mean plasma LDL cholesterol level was significantly (p<0.05) higher in the T_1_ and T_2_ groups in comparison to all the other treatment groups. There was no significant (p>0.05) difference in the mean plasma LDL cholesterol level among the treatment groups T_4_ and T_6_ and between T_3_, T_5_, and T_7_. The mean plasma LDL cholesterol level was significantly (p<0.05) lower in T_8_ group of broiler chickens in comparison to all other treatment groups.

The mean plasma HDL cholesterol level was significantly (p<0.05) lower in the T_1_ and T_2_ group of broiler chicken in comparison to all the other treatment groups. There were no significant differences in the mean plasma HDL cholesterol levels among the treatment groups T_4_, T_6_, and T_7_. The mean plasma HDL cholesterol level was significantly (p<0.05) highest in the T_8_ group of birds in comparison to all the other treatment groups.

### Plasma triglycerides

The mean plasma triglycerides level was significantly (p<0.05) higher in the T_1_ and T_2_ groups of broilers chicken in comparison to all the other treatments and controls. The lowest plasma triglycerides level was recorded in T_8_ group. The groups of birds which received the encapsulated bacteria (T_4_, T_6_, and T_8_) had significantly (p<0.05) lower mean total triglycerides as compared to the birds that supplemented with non-encapsulated bacteria (T_3_, T_5_, and T_7_).

## Discussion

### Effect on total proteins, albumin, and globulin

The result of the present study concurs with that of Siadati *et al.*, [12] who observed that probiotic supplementation increased the plasma protein and improved the growth performance in Japanese Quail. However, the results of the present study did not agree with findings of Li *et al*. [13] (*Lactobacillus sporogenes*) and Abdel-Hafeez *et al*. [14] who observed that the serum total protein concentration of birds supplemented with probiotic was significantly (p<0.05) lower than the control birds.

The lactic acid bacteria competitively exclude the pathogenic bacteria which reduce the breakdown of proteins to nitrogen and reduce the efficiency of dietary protein [15]. Thus, the utilization of amino acids and proteins is improved. Furthermore, increased villi height in the encapsulated probiotic-supplemented group as evident in the present study could have increased the protein absorption.

In the present study, the groups of birds which received the encapsulated bacteria either alone or in combination (T_4_, T_6_, and T_8_) had significantly (p<0.05) higher mean serum total protein, albumin, and globulin as compared to the birds that were not supplemented with any probiotic (T_1_ and T_2_) or supplemented with non-encapsulated bacteria (T_3_, T_5_, and T_7_).

### Effect on plasma total cholesterol, LDL cholesterol, and HDL cholesterol

The results of the present study concur with the findings of Siadati *et al*. [12], Ashayerizadeh *et al*. [16], and Iqramu *et al*. [17], who reported decreased serum total cholesterol level on supplementation of probiotic in poultry. However, the results of the present study did not agree with the findings of Shirisha *et al*. [18], who observed no significant difference in total cholesterol level between probiotic-supplemented birds and control birds.

The hypocholesterolemic effect observed in all the probiotic-supplemented broiler chicken may be due to mediated by various mechanisms. Lactic acid bacteria reduces the cholesterol by assimilating endogenous or exogenous originated cholesterol in the intestinal tract [19], reduces or inhibits the expression levels of Niemann-Pick C1-like 1 a protein, expressed on the surface of enterocytes, which reduces the cholesterol absorption [20]. Lactic acid bacteria produce bile salt hydrolase, enzyme which is responsible for deconjugation of bile salts and it helps to excrete more bile acids in the feces [21].

In our study too, mean plasma HDL cholesterol level was significantly (p<0.05) highest in the T_1_ and T_2_ group of broiler chicken in comparison to all the other treatments. There were no significant differences in the mean plasma HDL cholesterol level among the treatment groups T_4_, T_6_, and T_7_. The mean plasma HDL cholesterol level was significantly (p<0.05) lowest in the T_8_ group of birds in comparison to all the other treatment groups.

These results concur with the findings of Kalavathy *et al*. [22] who reported that probiotic supplementation decreased serum LDL level and but had no significant effect on serum HDL level broiler chickens. However, these findings did not agree with Ashayerizadeh *et al*. [16] who reported that supplementation of probiotic did not affect serum HDL and LDL concentration.

Supplementation of probiotic bacteria to broiler chicken altered the lipoprotein metabolism of birds favorably with more pronounced reduction on the total cholesterol and LDL cholesterol and increased HDL cholesterol concentration.

### Effect on plasma triglycerides

The results of the present study concur with the findings of Al-Saad *et al*. [23], Kalavathy *et al*. [22], Ashayerizadeh *et al*. [24], Abeer *et al*. [25], Iqramu *et al*. [17], and Ashayerizadeh *et al*. [16], who reported significant decrease in serum triglycerides level in broiler chickens on probiotic supplementation.

However, the results of the present study differ with the findings of Haddadin *et al*. [26], who reported no significant reduction in triglyceride level in serum and eggs on supplementation of *Lactobacillus acidophilus* in laying hens.

## Conclusion

Encapsulated bacteria either alone or in combination significantly increased total serum protein, albumin, and globulin values. Supplementation of the encapsulated bacteria either alone or in combination significantly lowered mean total cholesterol, LDL cholesterol, and triglycerides in comparison to the birds that were not supplemented with any probiotic or supplemented with non-encapsulated bacteria.

## Authors’ Contributions

PY, PS, and PV contributed to the planning and doing research work as study design and writing. PVK contributed to the nutritional aspects of the research. VC contributed for writing. All authors read and approved the final manuscript.

## References

[1] Lee S.H, Ingale S.L, Kim J.S, Kim K.H, Anushka L, Kim E.K, Kwon I.K, Kim Y.H, Chae B.J (2014). Effects of dietary supplementation with *Bacillus subtilis* LS 1–2 fermentation biomass on growth performance, nutrient digestibility, cecal microbiota and intestinal morphology of weanling pig. Anim. Feed. Sci. Tech.

[2] Zhang Z.F, Kim I.H (2014). Effects of multistrain probiotics on growth performance, apparent ileal nutrient digestibility, blood characteristics, cecal microbial shedding, and excreta odor contents in broilers. Poult. Sci.

[3] Kabir S.M.L (2009). The role of probiotics in the poultry industry. J. Mol. Sci.

[4] Vinderola C.G, Reinheimer J.A (2000). Enumeration of *Lactobacillus casei* in the presence of *L. acidophilus* bifidobacteria and lactic acid starter bacteria in fermented dairy products. Int. Dairy J.

[5] De Vos P, Faas M.M, Spasojevic M, Sikkema J (2010). Encapsulation for preservation of functionality and targeted delivery of bioactive food components. Int. Dairy J.

[6] Chavarri M, Maranon I, Ares R, Ibanez F.C, Marzo F, Villaran M.C (2010). Microencapsulation of a probiotic and prebiotic in alginate-chitosan capsules improves survival in simulated gastro-intestinal conditions. Int. J. Food Microbiol.

[7] Sharma A, Bhatia A, Singla R, Kaur G (2012). Improvement in bioactivity of *Lactobacillus* isolates by encapsulation in sodium alginate beads *In vitro*. Ann. Biol. Res.

[8] Herbert K, Kaplan L.A, Pesce A.J (1984). Lipids, in Clinical Chemistry: Theory. Analysis and Co-relation.

[9] Tietz N.W (1976). Fundamentals of Clinical Chemistry.

[10] McGowan M.W, Artiss J.D, Strandbergh D.R, Zak B (1983). A peroxidase-coupled method for the colorimetric determination of serum triglycerides. Clin. Chem.

[11] Lowry O.H, Rosebrough N.J, Farr A.L, Randall R.J (1951). Protein measurement with the folin phenol reagent. J. Biol. Chem.

[12] Siadati S.A, Ebrahimnezhad Y, Gh S.J, Shayegh J (2017). Evaluation of probiotic potential of some native *Lactobacillus* strains on the growth performance and serum biochemical parameters of Japanese quails (*Coturnix Coturnix japonica*) during rearing period. Bras. J. Poult. Sci.

[13] Li Y, Xu Q, Yang C, Yang X, Lv L, Yin C, Liu X, Yan H (2014). Effects of probiotics on the growth performance and intestinal microflora of broiler chickens. Pak. J. Pharm. Sci.

[14] Abdel-Hafeez H.M, Saleh E.S.E, Tawfeek S.S, Youssef I.M.I, Abdel-Daim A.S.A (2017). Effects of probiotic, prebiotic, and synbiotic with and without feed restriction on performance, hematological indices and carcass characteristics of broiler chickens. Asian Aust. J. Anim. Sci.

[15] Mikulec Z, Serman V, Mas N, Lukac Z (1999). Effect of probiotic on production results of fattened chickens fed different quantities of protein. Vet. Arhiv.

[16] Ashayerizadeh A, Dabiri N, Mirzadeh K.H, Ghorbani M.R (2011). Effect of dietary supplementation of probiotic and prebiotic on growth indices and serum biochemical parameters of broiler chickens. J. Cell Anim. Biol.

[17] Iqramu M.H, Nazim A, Mohammad A.M (2017). Comparative analysis of body weight and serum biochemistry in broilers supplemented with some selected probiotics and antibiotic growth promoters. J. Adv. Vet. Anim. Res.

[18] Shirisha R, Krishnadaida Raju M.V.L.N, Sai R.S, Ravinder R.V (2017). Effect of dietary supplementation of probiotic (problend) on Immune Status, biochemical profile and *E. coli* counts in commercial broiler chicken. J. Anim. Res.

[19] Gilliland S.E (1989). *Acidophilus* milk-products a review of potential benefits to consumers. J. Dairy Sci.

[20] Huang Y, Zheng Y (2010). The probiotic *Lactobacillus acidophilus* reduces cholesterol absorption through the down-regulation of Niemann-Pick C1-like 1 in Caco-2 cells. Br. J. Nutr.

[21] Surono I.S (2003). *In vitro* probiotic properties of indigenous dadhi lactic bacteria. Asian Aust. J. Anim. Sci.

[22] Kalavathy R, Abdullah N, Jalaludin S, Ho Y.W (2010). Effects of *Lactobacillus* cultures on growth performance, abdominal fat deposition, serum lipids and weight of organs of broiler chickens. Br. Poult. Sci.

[23] Al-Saad S, Abbod M, Abo Yones A (2014). Effect of some growth promoters on blood hematology and serum composition of broiler chickens. Int. J. Agric. Res.

[24] Ashayerizadeh O, Dastar B, Samadi F, Khomeiri M, Yamchi A, Zerehdaran S (2014). Comparison between the effects of two multi-strain probiotics and antibiotics on growth performance, carcass characteristics, gastrointestinal microbial population and serum biochemical values of broiler chickens. Sci. J. Anim. Sci.

[25] Abeer E.S.M, Mosaad S.A (2015). Effect of dietary probiotic and/or prebiotic supplementation on growth performance, carcass traits and some serum biochemical alterations in broiler chicken. J. Anim. Sci. Adv.

[26] Haddadin M.S.Y, Abdulrahim S.M, Hashlamoun E.A.R, Robinson R.K (1996). The effect of *Lactobacillus acidophilus* on the production and commercial composition of hen's egg. Poult. Sci.

